# RNAs as Proximity-Labeling Media for Identifying Nuclear Speckle Positions Relative to the Genome

**DOI:** 10.1016/j.isci.2018.06.005

**Published:** 2018-06-08

**Authors:** Weizhong Chen, Zhangming Yan, Simin Li, Norman Huang, Xuerui Huang, Jin Zhang, Sheng Zhong

**Affiliations:** 1 Department of Bioengineering, University of California San Diego, San Diego, CA 92093, USA; 2 Department of Pharmacology, University of California San Diego, San Diego, CA 92093, USA; 3 Division of Biological Sciences, University of California San Diego, San Diego, CA 92093, USA

**Keywords:** Genetics, Molecular Genetics, Data Analysis in Structural Biology

## Abstract

It remains challenging to identify all parts of the nuclear genome that are in proximity to nuclear speckles, due to physical separation between the nuclear speckle cores and chromatin. We hypothesized that noncoding RNAs including small nuclear RNA (snRNAs) and Malat1, which accumulate at the periphery of nuclear speckles (nsaRNA [nuclear speckle-associated RNA]), may extend to sufficient proximity to the genome. Leveraging a transcriptome-genome interaction assay (mapping of RNA-genome interactions [MARGI]), we identified clusters of nsaRNA-interacting genomic sequences (nsaPeaks). Posttranscriptional pre-mRNAs, which also accumulate to nuclear speckles, exhibited proximity to nsaPeaks but rarely to other genomic regions. Our combined DNA fluorescence *in situ* hybridization and immunofluorescence analysis in 182 single cells revealed a 3-fold increase in odds for nuclear speckles to localize near an nsaPeak than its neighboring genomic sequence. These data suggest a model that nsaRNAs are located in sufficient proximity to the nuclear genome and leave identifiable genomic footprints, thus revealing the parts of genome proximal to nuclear speckles.

## Highlights

• MARGI captures interactions of nuclear speckle-associated RNAs (nsaRNA) and DNA
• nsaRNA-interacting genomic sequences were clustered (nsaPeaks) in the genome
• Posttranscriptional pre-mRNAs and CDK9 proteins exhibited proximity to nsaPeaks
• Single-cell images confirmed proximity of nuclear speckles to an nsaPeak

## Graphical Abstract

**Figure:**
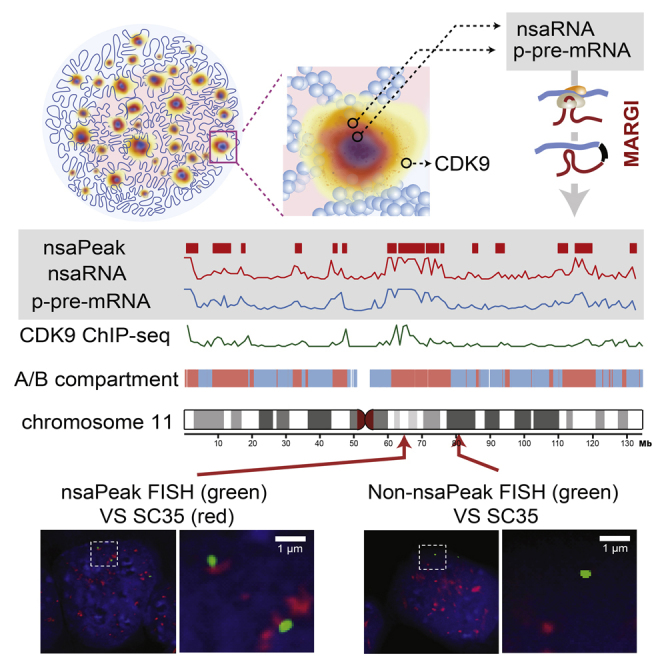

## Introduction

It is increasingly evident that positioning and organization of various subnuclear structures are critical for regulating gene expression, and therefore resolving the spatial organization of nuclear components has become a central task to nucleome research (Dekker et al., 2017). Nuclear bodies, previously known as interchromatin structures, typically exhibit non-overlapping spatial distributions with the genome (Brasch and Ochs, 1992, Sternsdorf et al., 1997). With an exception of nucleoli, which are positioned near ribosomal DNA (rDNA) (O'Sullivan et al., 2013), it remains challenging to identify the genomic sequences near most of the nuclear bodies, especially nuclear speckles (Lamond and Spector, 2003). Chromatin immunoprecipitation sequencing (ChIP-seq) targeting nuclear speckle core proteins rarely produces reproducible peaks (Kim et al., 2018), likely due to the lack of stable physical interactions between nuclear speckle core proteins and chromatin (Spector and Lamond, 2011, Chen, 2016, Kim et al., 2018).

**Figure 1:**
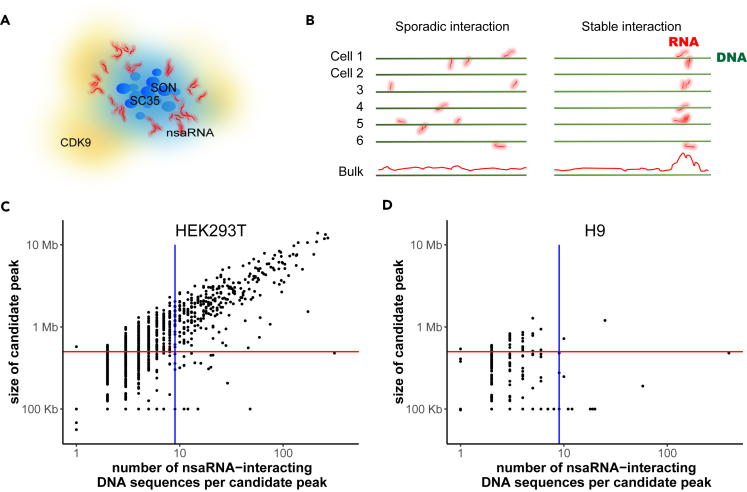
DNA Interaction Sites of nsaRNAs (A) A cartoon of multilayer structure of nuclear speckles. (B) Models of RNA-chromatin interaction in single cells, including sporadic interaction model and stable interaction model. (C and D) Candidate peaks of nsaRNA-interacting DNA sequences in the genome. The number of nsaRNA-interacting DNA sequences (x axis) is plotted against cluster size (y axis) for every candidate peak in HEK293T (C) and H9 hES cells (D). Vertical line, 9 reads; horizontal line, 500 Kb.

Advanced imaging technologies including super-resolution imaging have started to reveal the multilayer structure of nuclear speckles, with the proteins SC35 and SON at the center (Fei et al., 2017) and nuclear speckle-associated noncoding RNAs (nsaRNA) including small nuclear RNA (snRNA) and Malat1 (Fei et al., 2017) as well as posttranscriptional precursor mRNAs (pre-mRNA) accumulated at the periphery (Misteli and Spector, 1997, Cmarko et al., 1999). In addition, distribution of Cdk9-cyclin T1 complex correlates with nuclear speckles (Dow et al., 2010, Herrmann and Mancini, 2001) but more often extends beyond the periphery of nuclear speckles (Spector and Lamond, 2011) (Figure 1A). A number of other proteins are associated with nuclear speckles (Spector and Lamond, 2011); however, it remains unclear whether their distribution corresponds to specific layers. The microscopic observation that noncoding RNAs are located at the outer layer of nuclear speckles (Fei et al., 2017) led us to hypothesize that these peripheral noncoding RNAs may be present in sufficient proximity to nuclear genome, leaving identifiable proximal sequences as their genomic footprints. Hereafter, we call this hypothesis the “nsaRNA proximity” hypothesis.

The recent technology on global mapping of RNA-genome interactions (MARGI) enabled the identification of interacting genomic sequences of chromatin-interacting RNAs (Sridhar et al., 2017). After cross-linking and genome fragmentation, MARGI ligates RNA, a linker sequence, and proximal DNA to form an RNA-linker-DNA chimeric sequence, which is subsequently converted to double-stranded DNA and subjected to paired-end sequencing (see Figure 1 of Sridhar et al. (2017)). Because MARGI simultaneously assayed thousands of noncoding RNAs, including nsaRNAs, we will leverage MARGI data to test the nsaRNA proximity hypothesis.

Resolving spatial organization of nuclear components requires connecting information through different length scales and data types. Microscopic analyses have revealed non-uniform three-dimensional (3D) distribution of several types of RNAs in the nucleus. Prominent examples include Xist RNA cloud in adult female cells (Jonkers et al., 2008), accumulation of rRNA in nucleoli (Beven et al., 1996), and accumulation of snRNAs, Malat1, and posttranscriptional pre-mRNAs (p-pre-mRNAs) in nuclear speckles (Prasanth et al., 2010, Nakagawa et al., 2012, Tripathi et al., 2010, Galganski et al., 2017, Misteli and Spector, 1997, Cmarko et al., 1999, Girard et al., 2012). However, it remains a challenge to connect these microscopic findings with the latest information on 3D genome organization derived from genomics assays (Dekker et al., 2017). This challenge lies partially in the different length scales that vary in orders of magnitudes. For instance, the protein core of a nuclear speckle varies from one to several micrometers in diameter (Spector and Lamond, 2011), which is approximately 20%–50% of the spread of metaphase chromosomes (Lemke et al., 2002, Cremer and Cremer, 2001) or the diameters of chromosome territories (Cremer and Cremer, 2001, Bolzer et al., 2005). These relative sizes suggest that genomic regions in proximity to nuclear speckles may be significantly larger than the typical sizes of ChIP-seq or ATAC-seq peaks. Nevertheless, the enrichment of Xist RNA on X chromosome revealed by imaging (Jonkers et al., 2008) was successfully corroborated by genomics technologies including RAP-seq (Engreitz et al., 2014) and MARGI (Sridhar et al., 2017), offering an example of convergent findings from imaging and genomics approaches. In this work, we tested our “nsaRNA proximity” hypothesis by combining microscopic information and genomics data and aimed for establishing an RNA-based approach for identifying the relative positions of the folded genome and subnuclear structures.

## Results

### MARGI Captures Proximity of Nuclear rRNA to Ribosomal DNA

We used the co-localization of nuclear rRNA and rDNA (human rDNA complete repeating unit) in nucleoli (Beven et al., 1996) as a test bed system to verify the assumption that RNA-DNA ligation sequencing (MARGI) data reflect spatial co-localization of a group of nuclear body-associated RNAs with specific genomic sequences. We reanalyzed MARGI datasets from human embryonic kidney (HEK) cells (GEO: GSM2427902 and GSM2427903) and human embryonic stem (hES) cells (GEO: GSM2427895 and GSM2427896) (Sridhar et al., 2017), which yielded approximately 9.9 million and 5.6 million RNA-DNA sequence pairs, respectively (Table S1). To test whether rRNAs are enriched in the proximity of rDNA, we categorized the RNA-DNA sequence pairs by the RNA type (rRNA or other types) and by the DNA (rDNA or the rest of the genome [hg38]) (Table S1). Compared with the other types of RNA, rRNA exhibited more than 400-fold increase of odds to ligate with rDNA in HEK cells (odds ratio = 404, p value < 10^−16^) and more than 1,800-fold increase of odds in hES cells (odds ratio = 1,810, p value < 10^−16^), confirming that MARGI data reflected co-localization of nucleolus-associated RNA and DNA.

### nsaRNA-DNA Interaction Is Cell Type Specific

We asked which genomic regions are in proximity to nsaRNAs. At the single-cell level, there are four possible answers (models) to this question, which are (1) there is lack of nsaRNA expression; (2) nsaRNAs do not stably locate in the proximity of any specific genomic region in a single cell; (3) nsaRNAs are proximal to different DNA sequences in different single cells; however, none of these DNA sequences are shared by the majority of the cells; and (4) nsaRNAs are proximal to some DNA sequences and at least a fraction of these DNA sequences are shared by the majority of cells. Experiments with bulk cells could potentially differentiate the fourth model (stable interaction model) from its opposite (the first two models, collectively called the sporadic interaction model) (Figure 1B) but cannot further differentiate the first three models. Under the sporadic interaction model, bulk cell analysis (MARGI) is not expected to identify nsaRNA-DNA interactions (bulk lane, Figure 1B).

We used MARGI datasets to test the competing models. We reprocessed MARGI datasets generated from HEK and hES cells using the MARGI analysis pipeline (http://systemsbio.ucsd.edu/margi/) (Sridhar et al., 2017). This pipeline obtains the RNA-DNA read pairs with both ends uniquely mapped to the genome (hg38) and subsequently removes the proximal read pairs that may represent nascent transcripts. HEK and hES cells yielded 559,873 and 211,487 uniquely mapped RNA-DNA read pairs, respectively. In HEK cells, 14,904 pairs (2.5%) were nsaRNA-DNA pairs with the RNA end uniquely mapped to nsaRNAs (U1, U2, U4, U4atac, U5, U6, U6atac, U11, U12 [Carmo-Fonseca et al., 1992, Huang and Spector, 1992, Will and Luhrmann, 2011, Patel and Steitz, 2003, Pessa et al., 2008], 7SK [Peterlin et al., 2012, Prasanth et al., 2010], Malat1 [Tripathi et al., 2010]). In comparison, there were 1,857 nsaRNA-DNA pairs in hES cells, corresponding to only 0.88% RNA-DNA pairs in hES cells. Compared with HEK, hES-derived read pairs exhibited 3-fold reduction in odds of being nsaRNA-DNA repairs (odds ratio = 2.9, chi-square p value < 10^−16^), which is reminiscent of lack of nuclear speckle formation in hES cells, where SC35 proteins and nsaRNAs are diffusely distributed in the nuclei (Butler et al., 2009).

**Figure 2:**
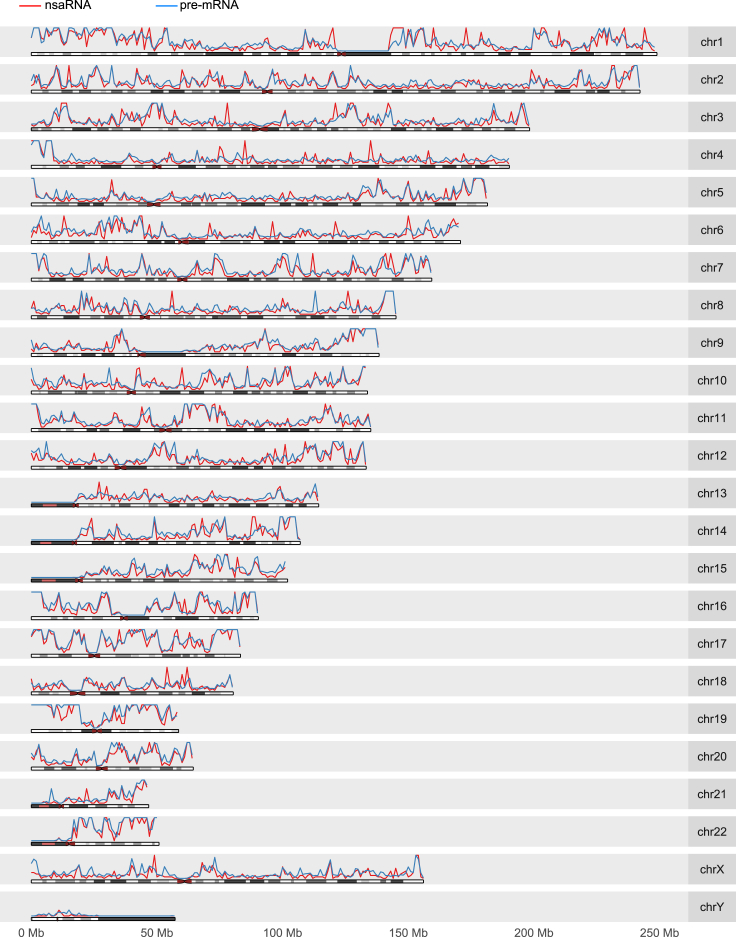
Genome-wide Density Distributions of nsaRNA-Interacting DNA Sequences (Red Curve) and Pre-mRNA-Proximal DNA (Blue Curve) in HEK293T Cells Binsize = 1 Mbp.

In HEK, the nsaRNA-interacting DNA formed candidate peaks (Figure 1C; red curve in Figure 2). Analysis with Homer (v4.8.3) yielded a total of 295 broad peaks (nsaPeaks, Figure 5), which contained 10,771 (72%) of the nsaRNA-interacting DNA sequences (permutation p value < 0.001). The sizes of nsaPeaks ranged from 100 kb to 13 Mb, on the same scale of nuclear lamina-associated domains that span 10 kb–10 Mb (van Steensel and Belmont, 2017). The clustering of nsaRNA-interacting DNA sequences in the genome is consistent with the stable interaction model. In contrast, nsaRNA-interacting DNA sequences barely exhibited any clustering formation in the genome of hES cells and yielded two broad peaks by Homer analysis. Adjusting for the total amount of candidate peaks and isolated nsaRNA-interacting DNA in each cell type (Figure 1D), hES cells exhibited more than 80-fold reduction in production of nsaRNA interaction peaks when compared with HEK cells (odds ratio = 88.9, p value < 10^16^). The sporadic distribution of nsaRNA-interacting DNA sequences in hES cells is also consistent with the lack of SC35 clusters in hES cells (sporadic interaction, Figure 1B).

We proceeded to test 295 nsaRNA-associated broad peaks (nsaPeaks) identified in HEK cells as the genomic regions close to nuclear speckles. We carried out these tests with two other types of nuclear speckle-associated molecules, namely, p-pre-mRNAs and CDK9 proteins.

### Posttranscriptional Pre-mRNAs Exhibit Spatial Proximity to nsaRNA-Interacting DNA

If nsaPeaks are near nuclear speckles, other nuclear speckle-associated molecules besides nsaRNAs may also exhibit enrichment in spatial proximity of nsaPeaks. Although splicing is generally initiated co-transcriptionally, not all splicing events are completed during transcription. The resulting p-pre-mRNAs are observed to cluster at the nuclear speckle domains (Misteli and Spector, 1997, Cmarko et al., 1999, Girard et al., 2012). The clustering of p-pre-mRNAs offers another characteristic of nuclear speckles. We leveraged this characteristic for testing nsaPeaks as the part of genome proximal to nuclear speckles. The key assumption of this test is that clustering of RNAs in 3D predicts clustering of their interacting genomic sequences in the genome. To test this assumption, we examined whether p-pre-mRNA-interacting genomic sequences exhibit clustering patterns or are sporadically distributed in the genome. We processed MARGI data from HEK cells (Sridhar et al., 2017) to identify p-pre-mRNA-DNA interactions. To identify pre-mRNA reads, we required the RNA end of a MARGI read pair to span across an exon-intron junction and cover the intron by 10 or more nucleotides. To eliminate the reads that were potentially derived from nascent pre-mRNAs, we removed any MARGI read pairs with the RNA end mapped within 2,000 nt to the genomic location where the DNA end was mapped to. The remaining 187,724 uniquely mappable sequence pairs representing p-pre-mRNA-DNA interactions were obtained. More than 93% (175,626) of these 187,724 read pairs represented interchromosomal interactions. The 187,724 DNA ends of these sequence pairs were not uniformly distributed in the genome (blue curve, Figure 2); instead they concentrated to certain genomic regions, yielding 284 broad peaks (Homer v4.8.3, broad peak option) (p value < 0.001, permutation test) (Figure S1). Taken together, p-pre-mRNAs exhibited proximity to remote DNA sequences. These remote interacting sequences exhibited clustering patterns in the genome, which corroborates with the idea that p-pre-mRNAs are clustered rather than diffusively distributed in the nucleus (Misteli and Spector, 1997, Cmarko et al., 1999, Girard et al., 2012).

We compared nsaRNA-interacting DNA and the DNA sequences proximal to p-pre-mRNAs by genome-wide density distributions, broad peaks, and genomic windows. The genome-wide distribution of p-pre-mRNA-proximal DNA sequences exhibited remarkable similarity to the distribution of nsaRNA-interacting DNA (Figure 2). A total of 170 (57.6%) nsaPeaks overlapped with p-pre-mRNA broad peaks (Figures S1 and S2A) (p value < 0.001, permutation test). Finally, we broke the genome into equal-sized windows and calculated the densities of nsaRNA-interacting DNA and p-pre-mRNA-proximal sequences in each window. These two density profiles exhibited a genome-wide correlation (Spearman correlation = 0.957, p value < 10^−16^) (Figures S2B and S2C). Taken together, p-pre-mRNA-proximal genomic regions exhibited significant overlap with nsaRNA-interacting DNA, supporting the idea that nsaPeaks reflect the parts of genome near nuclear speckles.

### Correspondence of Genome-wide Binding Profile of CDK9 and Genome-wide Distribution of nsaRNA-Interacting DNA

We compared genome-wide binding profile of CDK9 to genome-wide distribution of nsaRNA-interacting DNA sequences. ChIP-seq of nuclear speckle core proteins has been regarded a questionable approach for identifying the relative positions of nuclear speckles and the genome (Chen, 2016, Dekker et al., 2017), due to the physical separation of nuclear speckle cores from chromatin (Spector and Lamond, 2011). For example, suppose 95% of copies of a core protein, for instance, SC35, were located at the nuclear speckle cores and the other 5% were sporadically distributed, some of which are attached to chromatin, ChIP would select for the few chromatin-associated SC35 rather than those at the nuclear speckle cores. To alleviate this documented concern, we resorted to CDK9 proteins that are distributed throughout the core and periphery of nuclear speckles (Spector and Lamond, 2011, Dow et al., 2010, Herrmann and Mancini, 2001) for a ChIP-seq analysis. And even so we did not anticipate many overlaps between CDK9 ChIP-seq peaks and nsaRNA-interacting DNA sequences. We identified a total of 6,517 CDK9 peaks from HEK293T cells (GEO: GSM1249897) (Liu et al., 2013) (MACS2) (Zhang et al., 2008), of which only 551 (8.5%) were located within 200 bp of an nsaRNA-interacting DNA sequence. This overlap was statistically significant (p value < 0.001, permutation test), consistent with the idea that CDK9's distribution overlaps with nuclear speckles. However, the relatively small number of actual overlaps is reminiscent of the recognized challenge of using ChIP to identify nuclear speckle-interacting genomic regains (Chen, 2016, Dekker et al., 2017). These data also suggest that nsaPeaks do not precisely overlap with CDK9-bound promoters.

**Figure 3:**
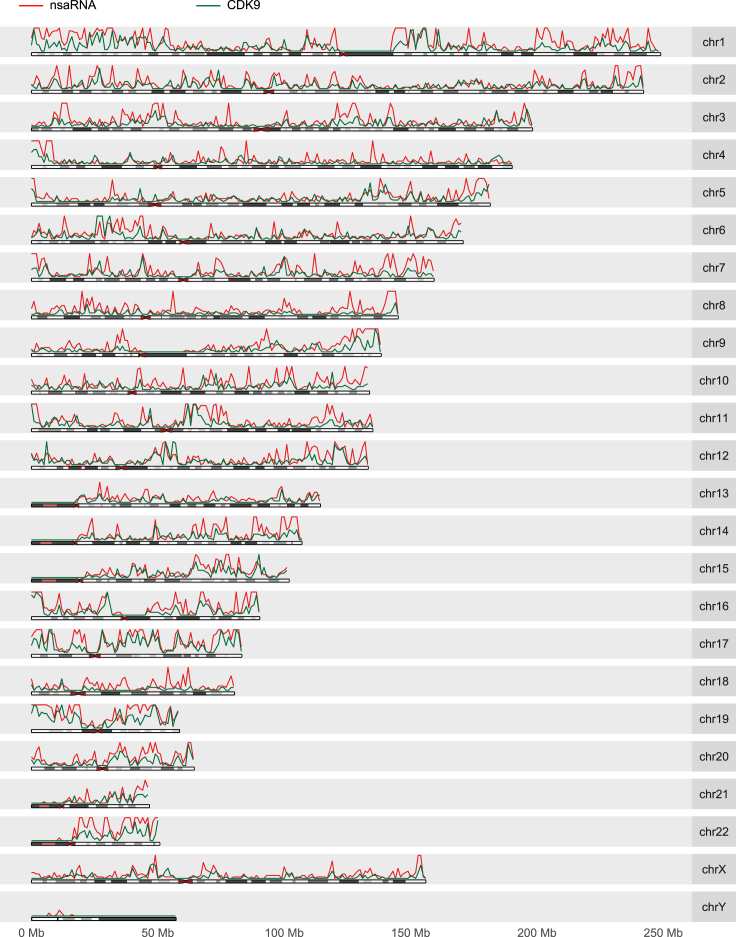
Genome-wide Density Distributions of nsaRNA-Interacting DNA Sequences (Red Curve) and CDK9 ChIP-seq Sequences (Green Curve) in HEK293T Cells Binsize = 1 Mbp.

Considering that the 3D distribution of CDK9 is centered at nuclear speckles (Dow et al., 2010, Herrmann and Mancini, 2001, Spector and Lamond, 2011), we tested the possibility that CDK9 ChIP-seq peaks cluster to the same genomic regions as nsaPeaks. Indeed, genome-wide density distribution of CDK9 peaks (green curve, Figure 3) resembled the density distribution of nsaRNA-interacting DNA (red curve, Figure 3). In a control comparison, genome-wide density distributions of H3K9me3 (Encode: ENCFF002AAZ) (Consortium, 2012) and nsaRNA-interacting DNA exhibited a poor correlation (Pearson correlation = 0.03, Spearman correlation = 0.27) (Figure S3). To test whether CDK9 binding sites cluster to the same genomic regions as nsaPeaks, we identified a total of 262 CDK9 broad peaks (sizes range from 514,083 bp to 6,262,520 bp, median size = 1,328,930 bp) (Homer, v4.8.3) (Heinz et al., 2010), of which 206 (78.6%) overlapped with nsaPeaks (p value < 0.001, permutation test) (Figures S4A and S5). Next, we split the genome (hg38) into 3.08 million 1,000-bp windows, of which 0.44 million windows overlapped with CDK9 broad peaks, of which 0.32 million windows also overlapped with nsaPeaks, suggesting strong association (odds ratio = 11.5, p value <10^−16^, Fisher's exact test) (Figure S4B). Taken together, although CDK9 does not frequently bind to the exact sequences as nsaRNA-interacting DNA, CDK9 binding sites accumulated to nsaPeaks, corroborating with the idea that nsaPeaks reflect the portion of genome closer to nuclear speckles.

### Co-localization of SC35 Clusters and nsaPeaks in Single Cells

**Figure 4:**
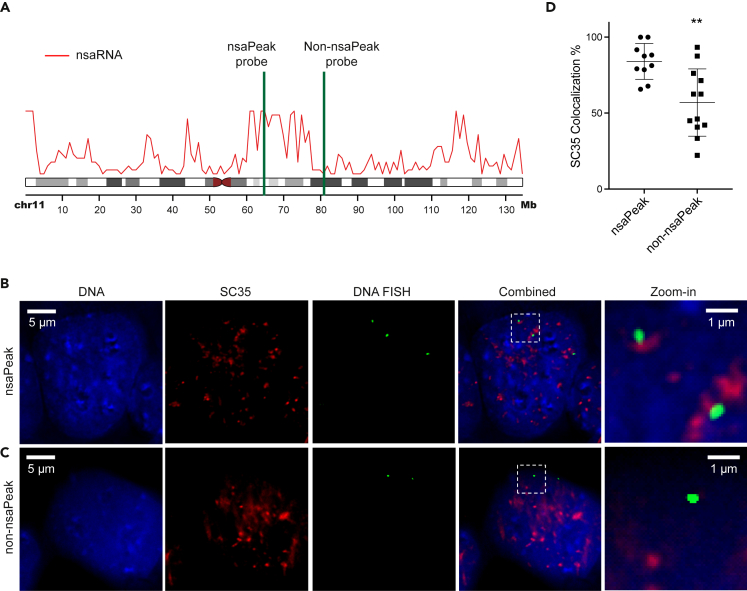
Visualization of Representative nsaPeak and Non-nsaPeak with SC35 Clusters (A) Genomic positions of nsaPeak probe and non-nsaPeak probe (green) with respect to nsaPeaks (red). (B and C) Representative images of HEK293T cells co-stained with Hoechst (DNA, blue), SC35 (red), and DNA FISH (green) with nsaPeak probe (B) and non-nsaPeak probe (C). Scale bar: 5 μm. Last column: zoom-in views of the selected regions in the dashed boxes. Scale bar: 1 μm. (D) The percentage of FISH spots that exhibited overlapping SC35 signal (y axis) in each image (dot) were plotted for samples interrogated with the nsaPeak probe images (left) and the non-nsaPeak probe (right). Error bar: SD. **p < 0.003.

We examined the proximity of nuclear speckles to nsaPeaks at single-cell resolution using a combination of immunofluorescence staining of a nuclear speckle core protein SC35 and DNA fluorescent *in situ* hybridization (FISH) (Sayegh et al., 2005). We opted to use commercially validated FISH probes, and we wanted the probes to be on the same chromosome arm. We identified a pair of probes satisfying these criteria on chromosome 11 with one probe (bacterial artificial chromosome plasmid DNA) inside an nsaPeak (Empire Genomics: RP11-772K10, hereafter called the nsaPeak probe) and the other probe outside the nsaPeaks (Empire Genomics: RP11-908J16, hereafter called the non-nsaPeak probe) (Figure 4A). We imaged 82 and 100 single cells with nsaPeak probe and non-nsaPeak probe, respectively, co-stained with SC35 antibody. Each cell exhibited 1 to 3 FISH spots, consistent with pseudotriploidy of HEK293T cells, and 20 to 35 SC35 clusters (Figures 4B and 4C).

To minimize the sensitivity of results to image analysis methods, we carried out two sets of analyses based on different analysis methods. First, we identified each FISH spot and its associated pixel on every z stack by particle analysis (ImageJ) (Schneider et al., 2012). A FISH spot was called isolated from SC35 clusters only when none of its associated pixels exhibited SC35 signal. Otherwise a FISH spot was called co-localized with SC35. This is a conservative approach to call isolated FISH spots. Among the 210 nsaPeak FISH spots identified from 82 individual cells, 170 FISH spots (84.0%) co-localized with SC35 clusters. In comparison, among the 193 non-nsaPeak FISH spots identified from 100 cells, 110 co-localized with SC35 (56.9%), reflecting a 3-fold reduction in odds (odds ratio = 3.1, p value < 5×10^−7^, chi-square test). We also summarized the proportion of co-localized FISH spots in each image. The 10 images stained with nsaPeak probe exhibited on average 84.0% of their FISH spots co-localized with SC35 (dots in left column, Figure 4D). In comparison, the 12 images (dots in right column) stained with the non-nsaPeak probe had on average 56.9% FISH spots co-localized with SC35 (p value < 0.003, t test) (Figure 4D).

In the second analysis, we compared the FISH-to-SC35 distance distributions between nsaPeak and non-nsaPeak samples. We computed center-to-center distance in 3D from every FISH spot to its nearest SC35 cluster. We summarized the number of center pairs at each distance from 1 to 10 voxels in every image (Figure S6). The nsaPeak images exhibited two to three times more center pairs than non-nsaPeak images at every distance (p value < 10^−5^, Kolmogorov test). For example, the nsaPeak images exhibited 1 to 18 center pairs at a distance of 8 voxels, whereas the non-nsaPeak images exhibited 0 to 3 at this distance (Figure S6). The different distance distributions suggest that the interrogated nsaPeaks are closer to the SC35 clusters than the interrogated non-nsaPeaks among the analyzed single cells. Taken together, the two analyses based on different analysis assumptions both revealed clear differences in relative positions of nuclear speckles to the two interrogated genomic regions. In summary, pre-mRNA data, CDK9 data, and single-cell image data supported the nsaPeaks as nuclear speckle-proximal genomic regions.

### nsaPeaks Correlate with the A Compartment in HEK Cells but Not in Embryonic Stem Cells

**Figure 5:**
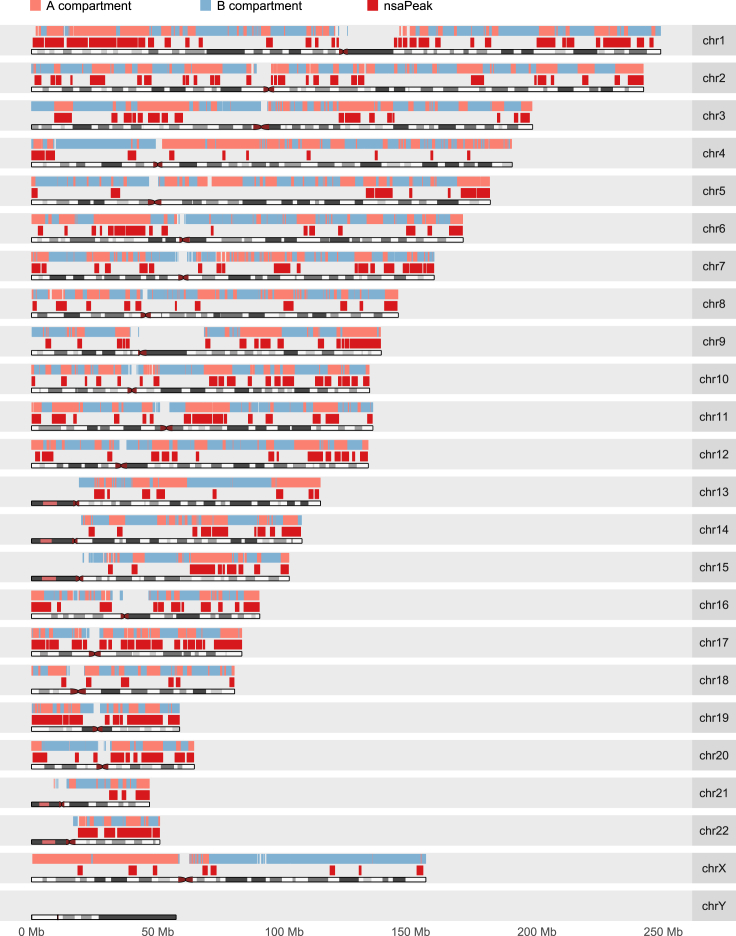
Genome-wide View of A (pink)/B (Light Blue) Compartments and nsaPeaks (Red)

We exploited how nsaPeaks fit into the current knowledge of the 3D structure of genome. Toward this goal, we compared nsaPeaks with nuclear compartments (Cockell and Gasser, 1999) and topologically associated domains (TADs) (Dixon et al., 2012). We called A/B compartments (Lieberman-Aiden et al., 2009) from HEK293T Hi-C data (Zuin et al., 2014) with Homer (v4.8.3) (Heinz et al., 2010, Lieberman-Aiden et al., 2009). Approximately half of the genome was associated with the A compartment (first row, Table S2). Approximately half of the genome in the A compartment and slightly more than 10% of the genome in the B compartment are associated with nsaPeaks (Figure 5), suggesting that nsaPeaks are enriched in but are not a complete subset of the genomic sequences in the A compartment. In line with this observation, nsaPeaks exhibited baseline increase of H3K4me3, H3K27ac, and H3K36me3 (25–75 quantiles, Figure S7B). In contrast, although a sizable proportion of the genome was categorized into the A compartment in hES cells (Dixon et al., 2015), merely two nsaPeaks were detected in hES cells (Figure 1D). These data argue against the possibility of the formation of nsaRNA clusters as a cause of A/B compartmentation of the nucleus, but suggest that nsaRNA clusters were formed in the same nuclear compartment that contains the A compartment of the genome.

### Genome Sequence in a TAD Tends to Be Either Entirely Close to or Distant from Nuclear Speckles

**Figure 6:**
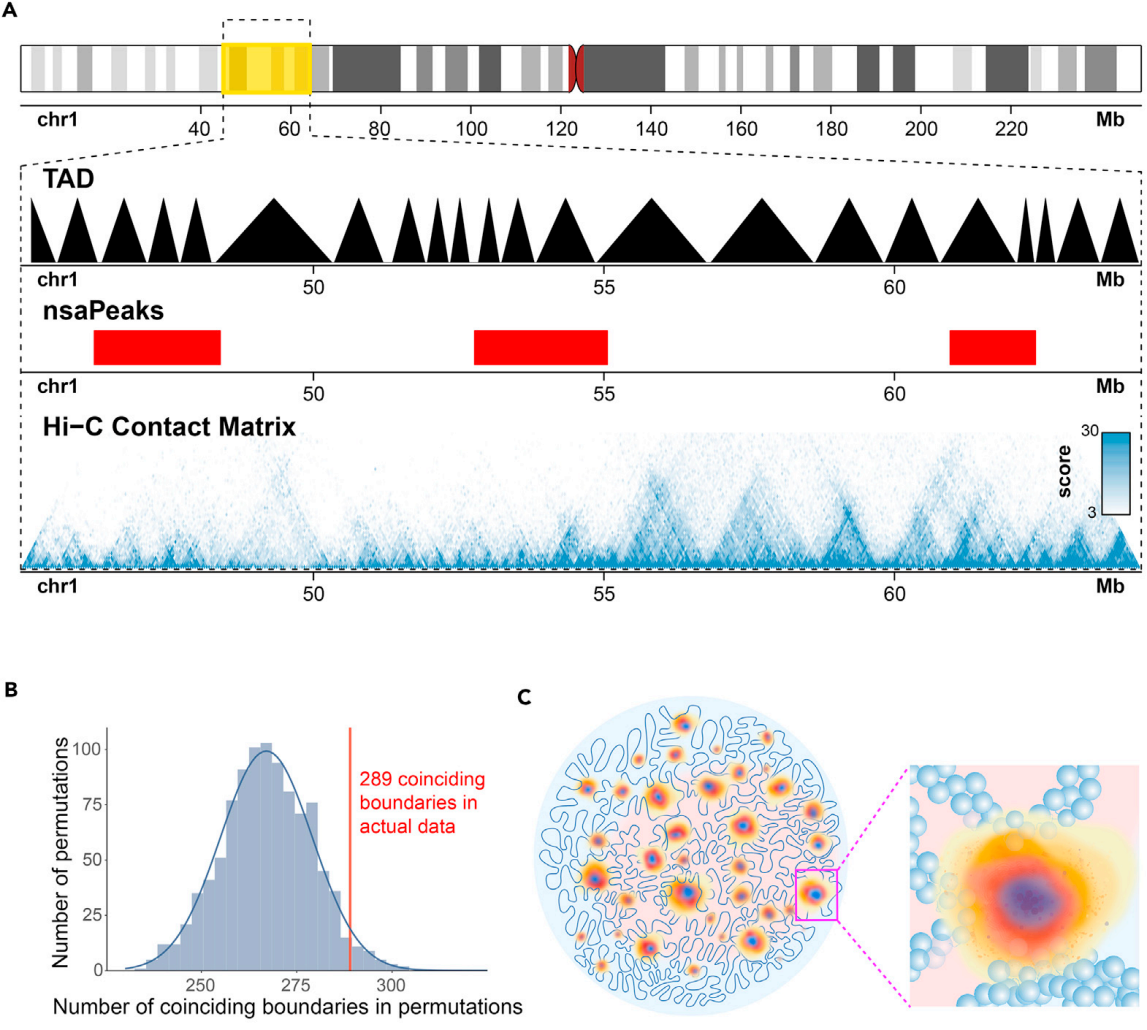
nsaPeaks and TADs (A) Genome view of TADs, nsaPeaks, and Hi-C contact matrix. (B) Background distribution of the numbers of TAD boundaries coinciding with TAD boundaries from 1,000 permutations (histogram and fitted curve) versus the number of observed coinciding boundaries in actual data (red line). (C) A model of boundaryless nuclear speckles and the genome. Nuclear speckle cores are in red. Other nuclear speckle-associated molecules exhibit diffusive patterns centered by nuclear speckle cores (blue, orange, yellow), some of which extend to sufficient proximity to certain TADs (balls, inset). Pink/light blue: A/B compartment.

The notion of TADs was derived from Hi-C experiments (Dixon et al., 2012), and TADs are subsequently proposed as a structural unit of genome organization (Dixon et al., 2016). We reasoned that organizational units should exhibit unity in relative positions to other nuclear components, and therefore proximity of the genome and nuclear speckles may offer an alternative test to this proposition. We compared the 3,258 TADs derived from HEK293T Hi-C data (GEO: GSM1081530) (Zuin et al., 2014) and nsaPeaks. Nearly 50% (289 of 590) of the boundaries of nsaPeaks were aligned with TAD boundaries (p value = 0.03, permutation test) (Figures 6A and 6B). A total of 74 nsaPeaks were aligned with 361 TADs, where each nsaPeak coincided with one TAD or several consecutive TADs (p value = 0.051, permutation test). Recognizing the sensitivity of peak boundaries to noises in data and to algorithm, we did another test with an alternative set of boundaries. Based on the significant overlap of nsaPeaks and CDK9 broad peaks (Figure S5), we merged the two sets of peaks (union) and obtained 334 union-peaks. Approximately 52% (350 of 668) of union-peak boundaries were aligned with TAD boundaries (p value = 0.001, permutation test). A total of 98 union-peaks were aligned with 468 TADs, where each union-peak coincided with one TAD or several consecutive TADs (p value = 0.005, permutation test). Taken together, MARGI data suggest that the genomic sequence of a TAD tends to be either entirely close to or entirely distant from nuclear speckles, supporting the proposition of TADs being structural units.

## Discussion

### Challenges in Identifying Relative Positions of Nuclear Speckles with Respect to Genomic Sequence

More than 150 proteins were reported to be associated with nuclear speckles (Saitoh et al., 2004), including small nuclear ribonucleoprotein particles and SR proteins essential for RNA splicing (Fu, 1995) and a number of kinases and phosphatases that regulate splicing machinery (Spector and Lamond, 2011). However, most of these proteins are not only present in nuclear speckles, and there is not sufficient data to assess the specificity of their localization to nuclear speckles. Therefore, the small number of proteins localized at the core of nuclear speckles, namely, SC35 and SON, received focal attention and were used as nuclear speckle markers in attempts to identify nuclear speckle-proximal genomic regions (Spector and Lamond, 2011, Chen, 2016, Kim et al., 2018). However, the detachment of nuclear speckle cores to chromatin suggested that ChIP-seq analyses of nuclear speckle core proteins would unlikely reveal the genomic sequences close to nuclear speckles (Chen, 2016). Thus, finding relative positions of nuclear speckles with respect to genomic sequences remains a major challenge in nucleome research (Dekker et al., 2017).

### RNAs as Media for Proximity Labeling

The increasing evidence on “noncoding RNAs functioning as scaffolds in the construction of nuclear bodies” points to the essential role of RNA in nuclear bodies (Wrighton, 2016). Nuclear speckles exhibit clear centers but showed inconsistent boundary lines when visualized by staining different nuclear speckle markers (Fei et al., 2017). Evidence of nsaRNA locating at the peripheral regions of nuclear speckles (Fei et al., 2017) fostered our hypothesis of this study that nsaRNAs serve as “proximity labeling” media, which “mark” proximal DNA (Figure 6C). Recently developed MARGI technology (Sridhar et al., 2017) enabled us to further examine this hypothesis by analyzing RNA-chromatin interactions of many noncoding RNAs at the same time.

### Cellular Heterogeneity and Assays of Bulk Cells

A rationale of ChIP-seq and ATAC-seq analyses of bulk cells is that the majority of single cells share the same transcription factor binding regions or transposase-accessible regions, and such commonality would be identified as peaks in bulk cell experiments. This rationale was verified by single-cell data produced by subsequently invented single-cell ChIP-seq (Rotem et al., 2015) and single-cell ATAC-seq technologies (Buenrostro et al., 2015). The same rationale is applicable to the MARGI technology in that only the genomic regions shared (relatively invariable) across many single cells would have a chance to appear in a bulk cell assay, whereas single-cell-specific interaction regions can hardly produce significant signals in a bulk cell assay (Figure 1B). Although there does not exist a single-cell version of MARGI technology, single-cell imaging analysis provided data consistent with this rationale.

### Genome as a Surrogate Coordinate for Studying Nuclear Organization

Revealing spatial organization of nuclear components has become a central task in nucleome research. This task is hindered by the lack of a 3D coordinate system for the nucleus. Without a coordinate system, spatial data obtained from different single cells cannot be aligned, making it difficult to derive or test for any underlying principles.

Chromosome territories fill sizable portions of interphase nuclei (Bolzer et al., 2005). The correspondence between the any piece of uniquely mappable sequence and its genomic location makes it possible for the nuclear genome to serve as a surrogate coordinate system of the nucleus, given that a 3D location in the nucleus could be approximated by its nearest genomic sequence. Compared with the alternative of not having any 3D coordinate at all, the genome-surrogate-3D coordinate provides a primitive means to record positional information that is potentially comparable across single cells or cell types. This surrogate coordinate has its own limitations, including lack of means to transform the surrogate coordinate into a physical coordinate and lack of power to differentiate chromosome pairs. This work was a test of this genome-surrogate-3D coordinate. Both chromosomes and nuclear bodies could have variable and cell-specific 3D positions; however, our data suggested that the relative positions between nuclear speckles and chromosomes were relatively stable. Thus, accumulated knowledge of relative positions of various nuclear components (van Steensel and Henikoff, 2000, van Steensel and Belmont, 2017) with respect to the nuclear genome may unleash the power of the genome-surrogate-3D coordinate in future analyses of spatial organization of the nucleus.

### Limited Resolution of MARGI-Derived nsaRNA-DNA Interactions

A major limitation of the proposed mapping strategy is the small number of MARGI-derived nsaRNA-DNA interaction read pairs. The total number of uniquely mapped nsaRNA-DNA read pairs in HEK293T cells from one MARGI experiment was 14,904. Each snRNA was only reflected by hundreds or thousands of read pairs, making it nearly infeasible to distinguish the proximity regions of major and minor spliceosomes. Another possible caveat of this analysis is the distance threshold for identifying p-pre-mRNA-DNA interactions. The currently applied threshold, 2,000 bp, may not be sufficient to remove all nascent RNA-DNA interactions. However, among the 187,724 identified p-pre-mRNA-DNA read pairs, the vast majority (175,626, 93%) were interchromosomal interactions. Therefore, even if the distance threshold is significantly increased, it is unlikely to result in large changes to the p-pre-mRNA analysis results.

## Methods

All methods can be found in the accompanying Transparent Methods supplemental file.

## Supplemental Information

Document S1. Transparent Methods, Figures S1–S7, and Tables S1 and S2
