# Supplementary material for: RNAs as Proximity-Labeling Media for Identifying Nuclear Speckle Positions Relative to the Genome
Source: iScience. 2018 Jun 8;4:204–15. doi: 10.1016/j.isci.2018.06.005 (PMC6146591; doi:10.1016/j.isci.2018.06.005)
Supplement: Document S1. Transparent Methods, Figures S1–S7, and Tables S1 and S2 [file mmc1.pdf]

ISCI, Volume *4*

Supplemental Information

RNAs as Proximity-Labeling Media for Identifying Nuclear Speckle Positions Relative to the Genome

Weizhong Chen, Zhangming Yan, Simin Li, Norman Huang, Xuerui Huang, Jin Zhang, and Sheng Zhong

Supplementary figures and legends

Figure S1. Genome-wide view of broad peaks of pre-mRNA proximal DNA (blue) and nsaPeaks (red), related to Figure 2.

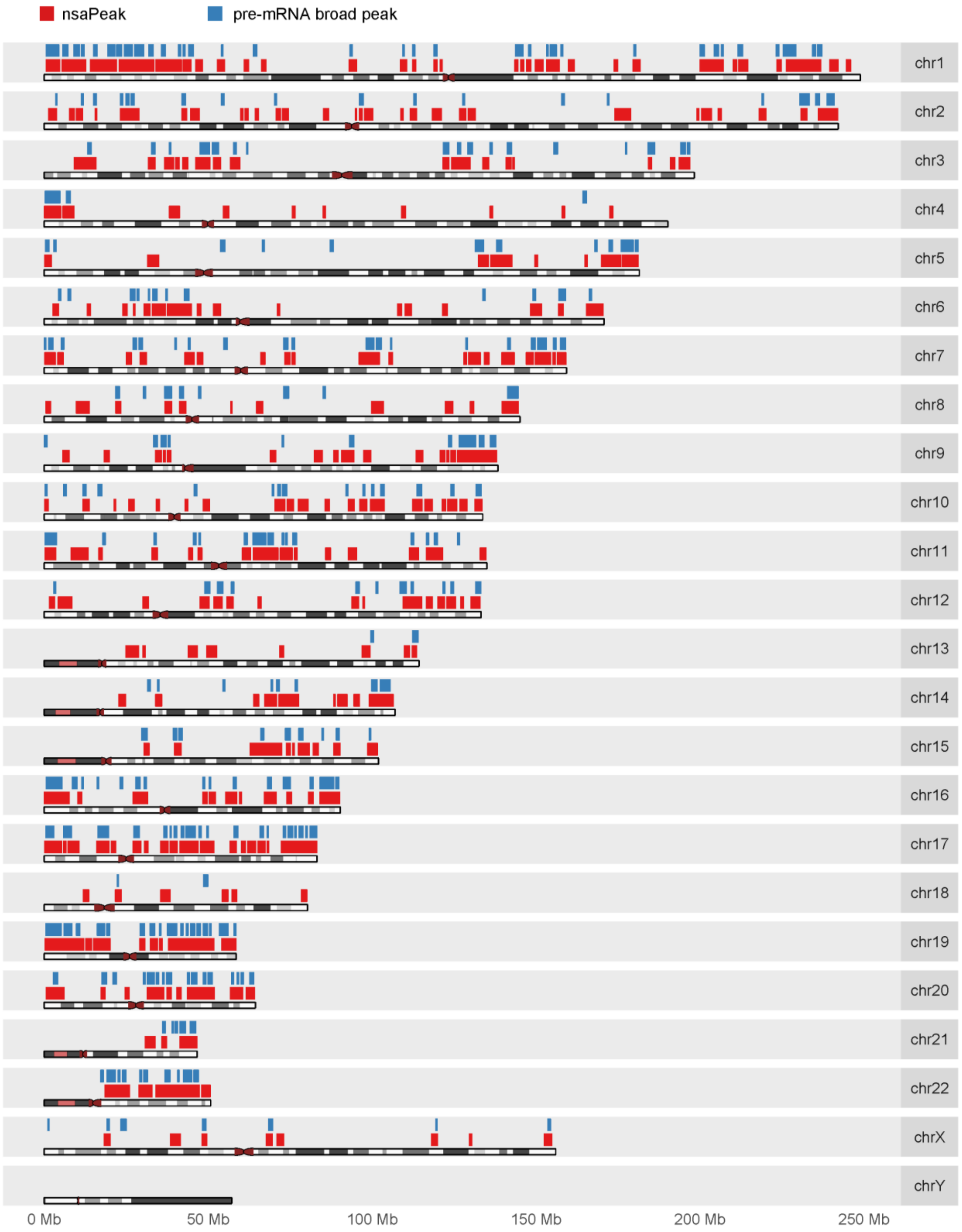

Figure S2. Comparison of nsaPeaks and pre-mRNA broad peaks, related to Figure 2. (A) Venn diagram of numbers of nsaPeaks (red), pre-mRNA broad peaks (blue), and overlaps. (B) Scatter plot of 3,102 genomic windows (1 Mb), with the number of nsaRNA interacting sequences (x axis) and the number of pre-mRNA proximal sequences in each window (y axis). (C) Scatterplot of 311 bins, where each bin is a group of 10 genomic windows, with the average number of nsaRNA interacting sequences (x axis) and the average number of pre-mRNA proximal sequences (y axis) of the 10 genomics windows of each bin.

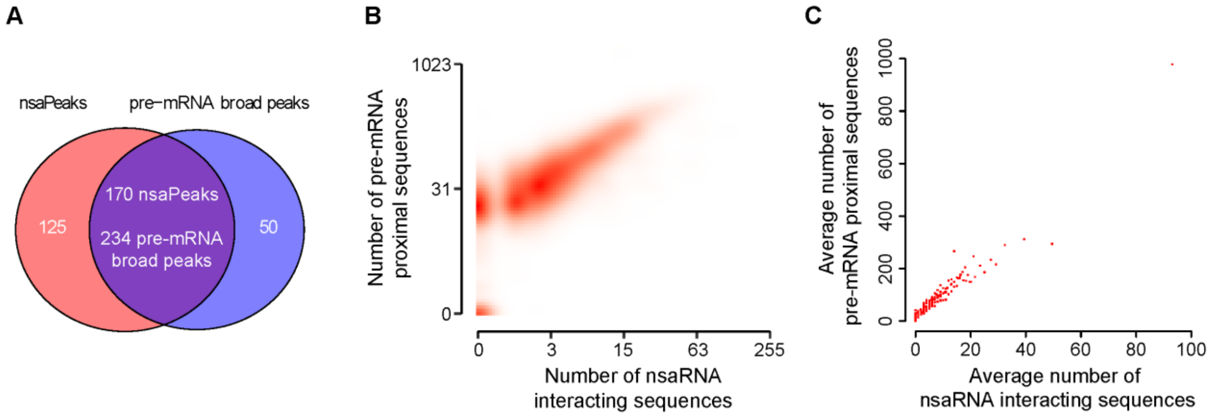

Figure S3. Genome-wide distribution of nsaRNA-interacting DNA sequences from MARGI (red) and H3K9me3 ChIP-seq sequences (blue) in HEK293T cells, related to Figure 3.

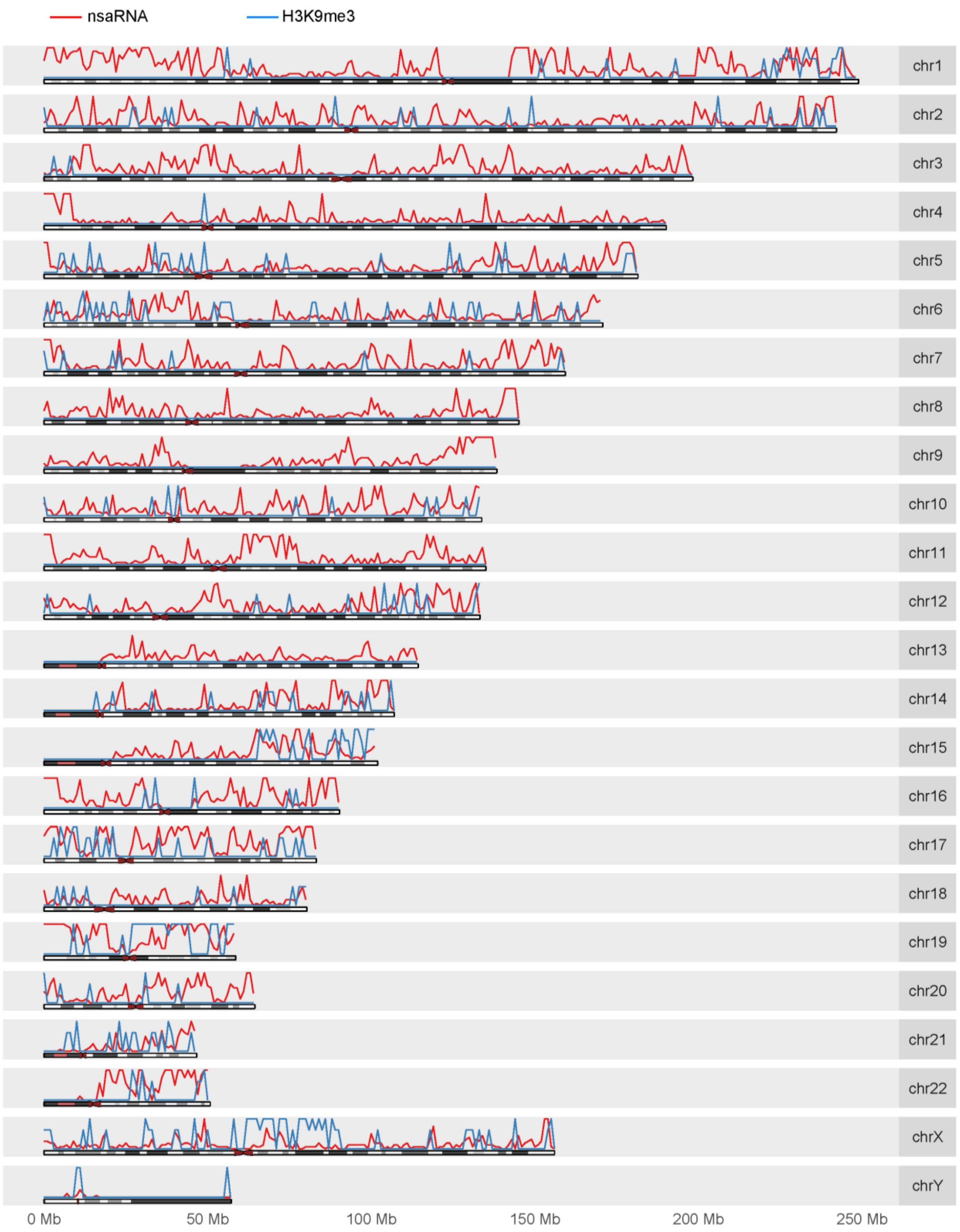

Figure S4. Comparison of nsaPeaks and CDK9 broad peaks, related to Figure 3. (A) Venn diagram. (B) Contingency table between genomic windows covered by CDK9 broad peaks and by nsaRNA-associated broad peaks. The genome (hg38) was split into 3,088,281 windows, with 1,000 bp equal size.

A

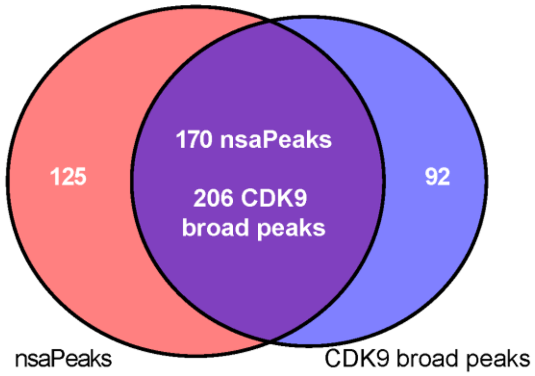

B

Odds ratio = 11.5. Fisher's exact test p-value < 10^{-16}.

		nsaPeaks		
		Inside	Outside	*Total*
CDK9 broad peaks	Inside	319,140	120,421	
	Outside	497,356	2,151,364	
	Total			3,088,281

Figure S5. Genome-wide view of CDK9 broad peaks (green) and nsaPeaks (red), related to Figure 3.

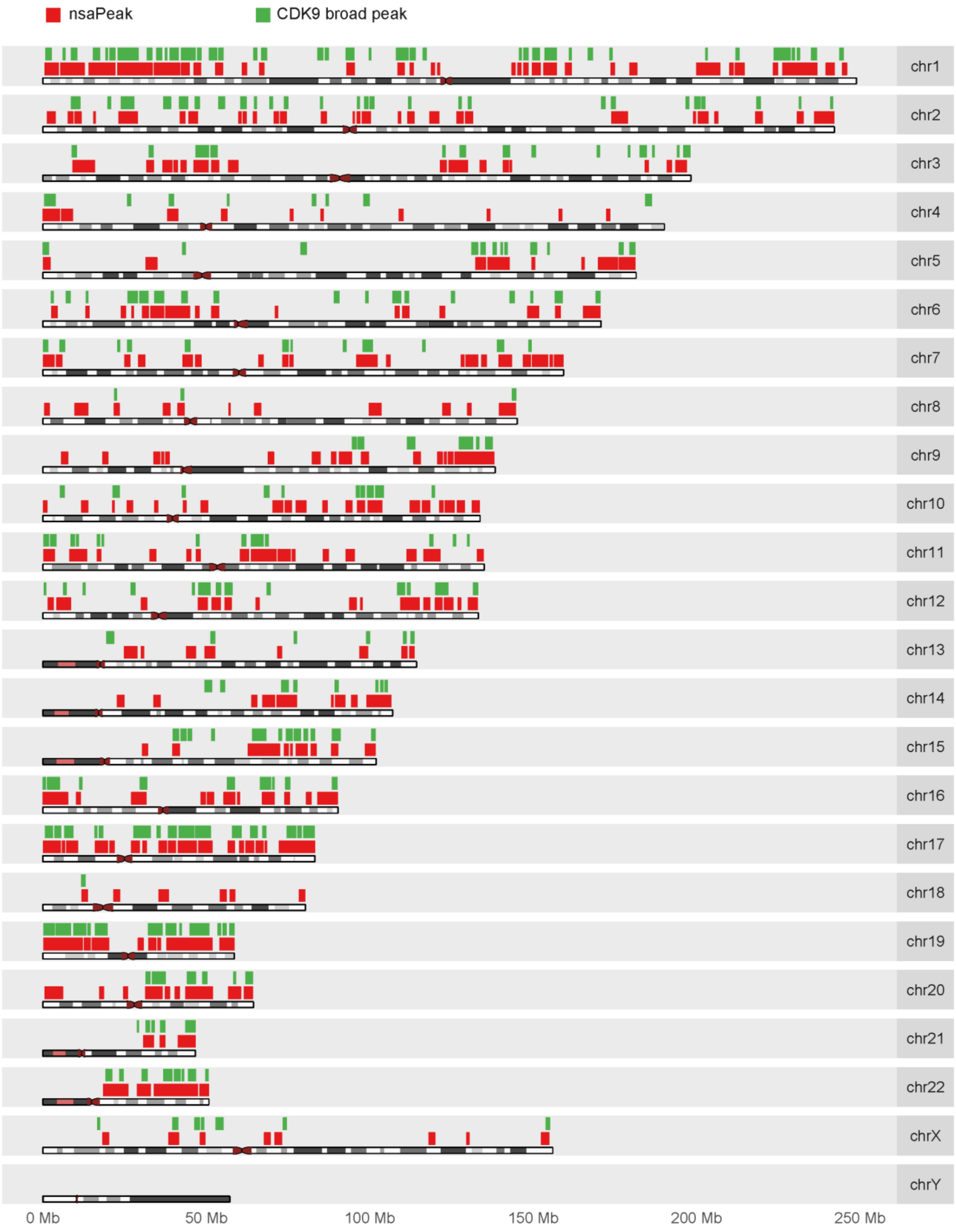

Figure S6. Distances between FISH spots and SC35 clusters, related to Figure 4. The center-to-center distance was calculated between each FISH spot and its nearest SC35 cluster. (A) The number of FISH spots (y axis) that have SC35 clusters at each designated distance (x axis) was plotted in each image (each dot) stained with the nsaPeak (black) or the non-nsaPeak probe (grey). There were 10 black dots and 12 grey dots in each column. (B) The average number of FISH spots that have SC35 clusters at each designated distance (x axis) in the 10 nsaPeak images (black) and 12 non-nasPeak images (grey). Error bar: 95% confidence interval.

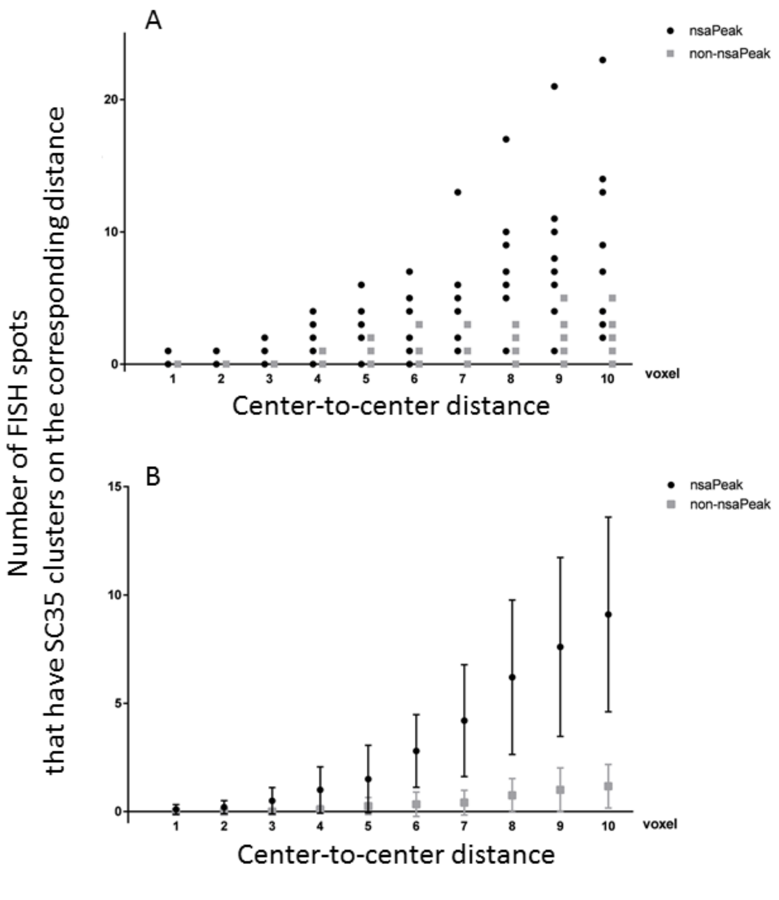

Figure S7. Gene expression (A) and histone modification levels (B) in nsaPeaks and the rest of the genome, related to Figure 5. (A) Violin plots of gene expression levels (y axis). A total of 21,566 and 15,386 genes were inside (nsaPeaks column) and outside of nsaPeaks (non-nsaPeak column), respectively. TPM: transcripts per million. (B) Distribution of the total length (bp) of histone modification ChIP-seq peaks in each 100,000bp genomic window (y axis), for the genomic windows inside (red) and outside nsaPeaks (green).

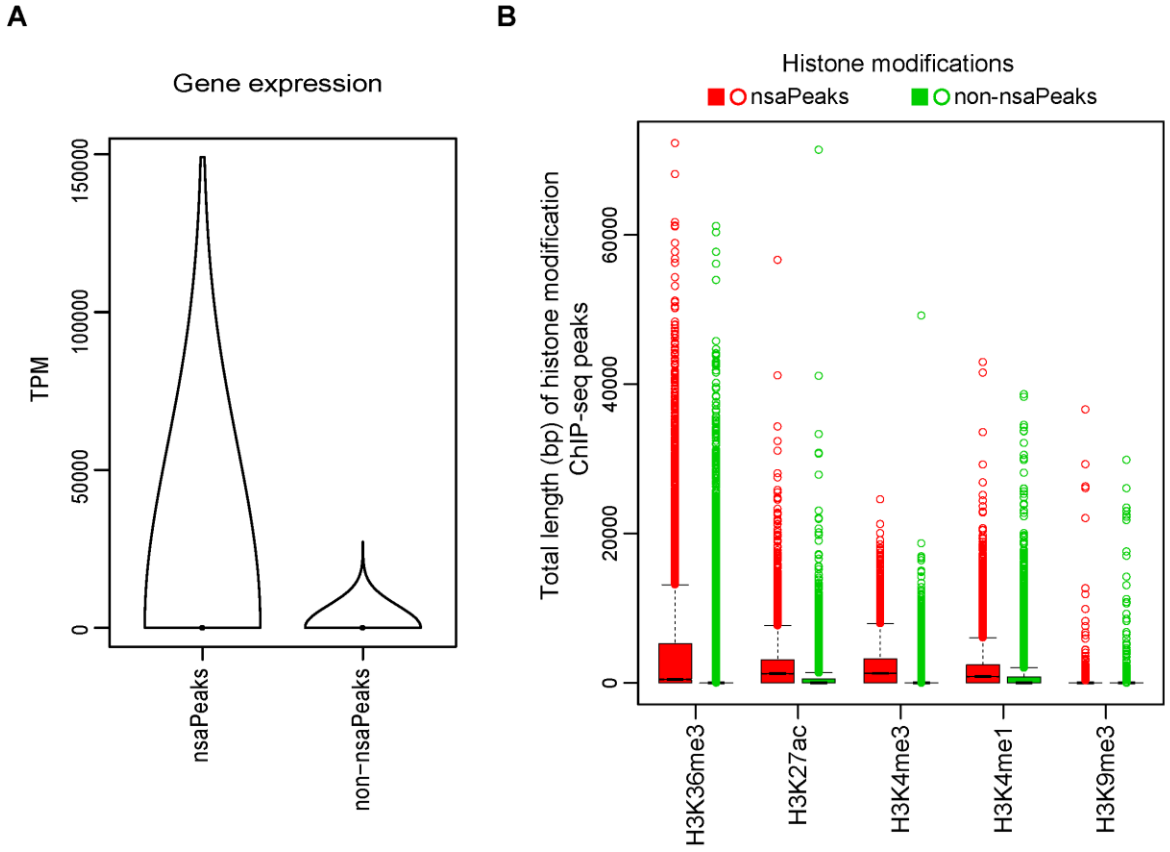

Supplementary tables

Table S1: Contingency table of RNA-DNA sequence pairs in HEK293T (A) and hES cells (B), related to Figure 1. Each pair is uniquely assigned to a cell based on its RNA-end sequence (columns) and DNA-end sequence (rows). rDNA: human ribosomal DNA complete repeating unit (Genbank ID: U13369.1), which is not assembled into the latest genome assembly (hg38). rRNA: transcripts originated from rRNA genes 5S, 5.8S, 28S, and 18S.

(A) HEK293T, odds ratio = 404, p-value $<10^{-16}$

RNA-end / DNA-end	rRNA	Other RNA
rDNA	132,469	18,984
hg38 DNA	167,417	9,604,768

(B) hES, odds ratio = 1,809, p-value $<10^{-16}$

RNA-end / DNA-end	rRNA	Other RNA
rDNA	809,763	29,366
hg38 DNA	71,531	4,681,715

Table S2. Contingency table between genomic windows covered by A compartment and by nsaPeaks, related to Figure 5. The genome (hg38) was split into 3,088,281 windows (with 1,000 bp equal size), among which 2750850 windows of either A or B compartment were taken for analysis. Odds ratio = 6.86. Fisher's exact test p-value < 10^{-16}.

	Inside nsaPeaks	*Outside nsaPeaks*	*Total*
A compartment	652,352	789,548	1,441,900
B compartment	140,711	1,168,239	1,308,950
Total	793,063	1,957,787	2,750,850

Transparent Methods

Datasets and accession numbers

Public datasets used in this work are MARGI data from HEK293T cells (GEO: GSM2427902 and GSM2427903) and H9 hES cells (GEO: GSM2427895 and GSM2427896) (Sridhar et al., 2017), CDK9 ChIP-seq (GEO: GSM1249897) (Liu et al., 2013) control ChIP-seq (GEO: GSM2423406) (Consortium, 2012) and Hi-C data from HEK293T cells (GEO: GSM1081530) (Zuin et al., 2014), RNA-seq (GEO: GSM2155552) (Ustianenko et al., 2016), H3K4me3 ChIP-seq (GEO: GSM945288, Encode: ENCFF001FJZ) and control ChIP-seq (GEO: GSM945256, Encode: ENCFF001HNC) from HEK293 cells (Thurman et al., 2012), H3K4me1 ChIP-seq (Encode: ENCFF002AAV) (Frietze et al., 2012), H3K9me3 ChIP-seq (Encode: ENCFF002AAZ) (Consortium, 2012), H3K27ac ChIP-seq (Encode: ENCFF002ABA) (Frietze et al., 2012), H3K36me3 ChIP-seq (Encode: ENCFF002ABD) (Consortium, 2012), control ChIP-seq (GEO:GSM935586, Encode: ENCFF000WXY) (Consortium, 2012) from HEK293 cells.

Mapping MARGI data

After removing RCR duplicates, the RNA-end and the DNA-end of a read pair were separately mapped to the genome (hg38) using STAR (Version 2.5.1b) (Dobin et al., 2013). Splice junction was allowed in mapping the RNA-end, by feeding the junction information (gtf file from ENSEMBL, hg38 release 84) to STAR. Splice junction was not allowed in mapping the DNA-end. Only the read pairs with both the RNA-end and the DNA-end uniquely mapped to the genome were used for downstream analysis.

Identifying rRNA-DNA read pairs

Human rRNA genes include 45S (18S, 5.8S and 28S) in rDNA (human ribosomal DNA complete repeating unit, GenBank: U13369.1) as well as 5S and 5.8S in the human genome assembly (hg38) (Stults et al., 2008). A MARGI read pair is categorized as an rRNA-DNA pair when the RNA-end is uniquely mapped to any human rRNA gene and the DNA-end is uniquely mapped to a combined “genome” of hg38 concatenated with rDNA.

Identifying nsaRNA-DNA read pairs

Human U1, U2, U4, U4atac, U5, U6, U6atac, U11, U12, 7SK, and Malat1 genes are considered nsaRNA genes. A MARGI read pair is categorized as an nsaRNA-DNA pair when the RNA-end is uniquely mapped to any human rRNA gene and the DNA-end is uniquely mapped to human genome (hg38). To minimize inclusion of nascent transcripts, the read pairs with the RNA-end and DNA-end mapped to within 2,000 bp in the genome are removed from further analysis.

Identifying pre-mRNA-DNA pairs

A MARGI read pair is categorized as a pre-mRNA-DNA pair when the RNA-end is uniquely mapped to an exon-intron junction with at least 10 bp overlap with the intron and the DNA-end is uniquely mapped to human genome (hg38). To minimize inclusion of nascent transcripts, the read pairs with the RNA-end and DNA-end mapped to within 2,000 bp in the genome are removed from further analysis.

Calling peaks and broad peaks

ChIP-seq and control ChIP-seq reads were mapped to human genome (hg38) and the uniquely mapped reads were fed to MACS2 (Zhang et al., 2008) to call peaks. CDK9 broad peaks, pre-mRNA broad peaks, and nsaPeaks were identified by the findPeaks module in Homer (v4.8.3) (Heinz et al., 2010). Any nsaPeak containing less than 9 MARGI reads was removed from further analysis.

Calling TADs and A/B compartments

HEK293 Hi-C data (GEO: GSM1081530) (Zuin et al., 2014) were aligned to hg38 retaining uniquely mapped reads. TADs were identified using a previously described HMM model (Dixon et al., 2012) automated in the GITAR software (Calandrelli et al., 2018). A/B compartments were called by the runHiCpca module in Homer (v4.8.3) (Heinz et al., 2010).

DNA FISH and immunofluorescence staining

The nsaPeak probe (RP11-772K10, covering chr11:64,663,168-64,947,112) with 5-ROX conjugate and the non-nsaPeak probe (RP11-908J16, covering chr11:80,767,575-80,980,051) with fluorescein conjugate were ordered from Empire Genomics. HEK293T cells were used through this study. In each experiment, cells were seeded on 18 X 18 mm glass coverslips with #1.5 thickness (#12-541A, Fisher Scientific) in 6-well tissue culture plate (Thermo Fisher Scientific) and grown in DMEM high-glucose media containing 10% (v/v) fetal bovine serum and 1% (v/v) penicillin-streptomycin at 37°C with 5% CO_2. Once reaching approximately 80% confluency, the cells were rinsed with PBS and fixed with 4% paraformaldehyde (PFA) in pH 7.2 phosphate-buffered saline (PBS) for 30 min at room temperature. PFA was discarded and residual PFA was quenched by incubation with 0.1 M Tris buffer (pH 7.4) at room temperature for 10 min followed with one wash with PBS. Cells were permeablized with PBS containing 0.1% saponin (#84510-100, Sigma) and 0.1% TritonX-100 for 10 min, then with 20% glycerol in PBS for 20 min at room temperature with gentle shaking. Cells were rapidly frozen in liquid nitrogen and thawed at room temperature for three cycles, and rinsed with PBS. To detect SC35, cells were blocked with 5% bovine serum albumin (BSA) in PBS with

0.1% TritonX-100 (PBST) at 37°C for 30 min, and incubated with mouse monoclonal anti-SC35 antibody (1:250) (#Ab11826, Abcam, RRID: AB_298608) in blocking buffer at 37°C for 1 hour. Cells were washed with PBST for 10 min for twice with gentle shaking, incubated with goat anti-mouse IgG antibody conjugated with Alexa647 (1:200 dilution) (#A21236, Invitrogen, RRID: 141725) in blocking buffer at 37°C for 30 min, and then washed again with PBST for 10 minutes twice while shaking. The cells were fixed again with 2% PFA at room temperature for 10 min, quenched with 0.1 M Tris buffer as previously described and washed with PBS for 5 min. Cells were incubated with 0.1 M HCl for 30 min at room temperature, followed by 1 hr incubation with 3% BSA and 100 μg/mL RnaseA (#EN0531, Thermo Fisher Scientific) in PBS at 37°C. Cells were permeablized again with 0.5% saponin and 0.5% TritonX-100 in PBS for 30 min at room temperature with gentle shaking, and rinsed with PBS. Cells were further denatured by incubation in 70% formamide with 2X saline-sodium citrate (SSC) buffer at 73°C for 2 min 30 sec and then incubation in 50% formamide with 2X SSC at 73°C for 1 min. For each coverslip, 1.2 μL of FISH probes were mixed with 4.8 μL formamide, incubated at 55°C for 15 min and mixed with 6 μL 2X hybridization buffer (8X SSC with 40% dextran sulfate) followed with denaturation at 75°C for at least 5 min until the cells were ready. 12 μL of FISH probe mixture was added onto a glass slide and quickly covered by freshly denatured coverslips with the cell side facing down. The coverslip was sealed with rubber cement and incubated in a humidified chamber at 37°C for 24 hr in the dark. The coverslips were collected the next day, washed twice with 50% formamide with 2X SSC for 15 min each at 37°C, three times with 2X SSC for 5 min each at 37°C, three times with 4X SSC containing 0.1% Tween 20 for 5 min each at room temperature, with gentle shaking, and rinsed with PBS. Cells were then stained with Hoescht 33342 (1:500 dilution) (#62249, Thermo Fisher Scientific) for 15 min followed with 5 min washing in PBS, mounted on slides with 80% glycerol in PBS and sealed with nail polish. Images in size of 1024 X 1024 were acquired on wide-field SIM DeltaVision Deconvolution Microscope using a 100X/1.40 oil objective (GE Healthcare Life Sciences) (pixel size = 0.66 μm). A series of z-stack images across the cells were acquired with thickness of 0.15 μm. Deconvolution was performed on these Z-stacks for subsequent image analysis.

Co-localization analysis

Deconvoluted images for each field of view contain a series of z stacks in three channels, DAPI, FISH and SC35. FISH spots were identified by performing particle analysis on the 2D maximal projection of z stacks of each field of view in the FISH channel with the threshold being set to the minimal value allowing only FISH spots to be recognized as “particles” in the size range of 10 – 500 pixels. These FISH particle regions were saved and applied to the z-stacks of FISH and SC35 channels and z-axis profiles of selected regions (min, max and mean values of fluorescence intensity) in both channels were recorded and examined. Positive co-localization of a given FISH

spot with SC35 was defined by the presence of positive SC35 signals above background in any of the FISH-signal containing regions of that FISH spot. In order to determine one FISH region as positive SC35-colocalized, it needs to contain more than one stack with mean intensity above SC35 background, or contain more than half amount of stacks with max intensity over SC35 background. For each analyzed image, SC35 background value was based on the average mean intensities in areas outside of SC35 clusters within the nucleus region. For each field of view, the SC35 co-localization rate represents the ratio of the amount of SC35-colocalized FISH spots over the amount of total FISH spots.

Center-to-center distances were calculated as follows. After deconvolution, each cluster or spot was identified as a connected 3D region such that all voxels within this region are above a threshold. The threshold was determined as described previously (Raj et al., 2008). Briefly, each deconvoluted image was scanned to identify all pixels on every stack that was could not possibly be background. The threshold was chosen such that the number of detected fluorescent clusters would not change within 3% variation of this threshold. The center of a cluster (spot) was calculated as the gravity center. Center-to-center distance was calculated with voxel as the unit.

Supplemental references

CALANDRELLI, R., WU, Q., GUAN, J. & ZHONG, S. 2018. GITAR: An open source tool for analysis and visualization of Hi-C data. *bioRxiv,* https://doi.org/10.1101/259515.

CONSORTIUM, E. P. 2012. An integrated encyclopedia of DNA elements in the human genome. *Nature,* 489**,** 57-74.

DIXON, J. R., SELVARAJ, S., YUE, F., KIM, A., LI, Y., SHEN, Y., HU, M., LIU, J. S. & REN, B. 2012. Topological domains in mammalian genomes identified by analysis of chromatin interactions. *Nature,* 485**,** 376-80.

DOBIN, A., DAVIS, C. A., SCHLESINGER, F., DRENKOW, J., ZALESKI, C., JHA, S., BATUT, P., CHAISSON, M. & GINGERAS, T. R. 2013. STAR: ultrafast universal RNA-seq aligner. *Bioinformatics,* 29**,** 15-21.

FRIETZE, S., WANG, R., YAO, L., TAK, Y. G., YE, Z., GADDIS, M., WITT, H., FARNHAM, P. J. & JIN, V. X. 2012. Cell type-specific binding patterns reveal that TCF7L2 can be tethered to the genome by association with GATA3. *Genome Biol,* 13**,** R52.

HEINZ, S., BENNER, C., SPANN, N., BERTOLINO, E., LIN, Y. C., LASLO, P., CHENG, J. X., MURRE, C., SINGH, H. & GLASS, C. K. 2010. Simple combinations of lineage-determining transcription factors prime cis-regulatory elements required for macrophage and B cell identities. *Mol Cell,* 38**,** 576-89.

LIU, W., MA, Q., WONG, K., LI, W., OHGI, K., ZHANG, J., AGGARWAL, A. & ROSENFELD, M. G. 2013. Brd4 and JMJD6-associated anti-pause enhancers in regulation of transcriptional pause release. *Cell,* 155**,** 1581-1595.

RAJ, A., VAN DEN BOGAARD, P., RIFKIN, S. A., VAN OUDENAARDEN, A. & TYAGI, S. 2008. Imaging individual mRNA molecules using multiple singly labeled probes. *Nat Methods,* 5**,** 877-9.

SRIDHAR, B., RIVAS-ASTROZA, M., NGUYEN, T. C., CHEN, W., YAN, Z., CAO, X., HEBERT, L. & ZHONG, S. 2017. Systematic Mapping of RNA-Chromatin Interactions In Vivo. *Curr Biol,* 27**,** 610-612.

STULTS, D. M., KILLEN, M. W., PIERCE, H. H. & PIERCE, A. J. 2008. Genomic architecture and inheritance of human ribosomal RNA gene clusters. *Genome Res,* 18**,** 13-8.

THURMAN, R. E., RYNES, E., HUMBERT, R., VIERSTRA, J., MAURANO, M. T., HAUGEN, E., SHEFFIELD, N. C., STERGACHIS, A. B., WANG, H., VERNOT, B., GARG, K., JOHN, S., SANDSTROM, R., BATES, D., BOATMAN, L., CANFIELD, T. K., DIEGEL, M., DUNN, D., EBERSOL, A. K., FRUM, T., GISTE, E., JOHNSON, A. K., JOHNSON, E. M., KUTYAVIN, T., LAJOIE, B., LEE, B. K., LEE, K., LONDON, D., LOTAKIS, D., NEPH, S., NERI, F., NGUYEN, E. D., QU, H., REYNOLDS, A. P., ROACH, V., SAFI, A., SANCHEZ, M. E., SANYAL, A., SHAFER, A., SIMON, J. M., SONG, L., VONG, S., WEAVER, M., YAN, Y., ZHANG, Z., ZHANG, Z., LENHARD, B., TEWARI, M., DORSCHNER, M. O., HANSEN, R. S., NAVAS, P. A., STAMATOYANNOPOULOS, G., IYER, V. R., LIEB, J. D., SUNYAEV, S. R., AKEY, J. M., SABO, P. J., KAUL, R., FUREY, T. S., DEKKER, J., CRAWFORD, G. E. & STAMATOYANNOPOULOS, J. A. 2012. The accessible chromatin landscape of the human genome. *Nature,* 489**,** 75-82.

USTIANENKO, D., PASULKA, J., FEKETOVA, Z., BEDNARIK, L., ZIGACKOVA, D., FORTOVA, A., ZAVOLAN, M. & VANACOVA, S. 2016. TUT-DIS3L2 is a mammalian surveillance pathway for aberrant structured non-coding RNAs. *EMBO J,* 35**,** 2179-2191.

ZHANG, Y., LIU, T., MEYER, C. A., EECKHOUTE, J., JOHNSON, D. S., BERNSTEIN, B. E., NUSBAUM, C., MYERS, R. M., BROWN, M., LI, W. & LIU, X. S. 2008. Model-based analysis of ChIP-Seq (MACS). *Genome Biol,* 9**,** R137.

ZUIN, J., DIXON, J. R., VAN DER REIJDEN, M. I., YE, Z., KOLOVOS, P., BROUWER, R. W., VAN DE CORPUT, M. P., VAN DE WERKEN, H. J., KNOCH, T. A., VAN, I. W. F., GROSVELD, F. G., REN, B. & WENDT, K. S. 2014. Cohesin and CTCF differentially affect chromatin architecture and gene expression in human cells. *Proc Natl Acad Sci U S A,* 111**,** 996-1001.
